# Metal Complexes or Chelators with ROS Regulation Capacity: Promising Candidates for Cancer Treatment

**DOI:** 10.3390/molecules27010148

**Published:** 2021-12-27

**Authors:** Xiang Li, Yuhui Wang, Man Li, Huipeng Wang, Xiongwei Dong

**Affiliations:** 1 College of Chemistry and Molecular Engineering, Peking University, Beijing 100871, China; 2 School of Chemistry, Central China Normal University, Wuhan 430079, China; wangyuhui1997@mails.ccnu.edu.cn (Y.W.); liman000000@mails.ccnu.edu.cn (M.L.); 3 National Local Joint Engineering Laboratory for Advanced Textile Processing and Clean Production, Wuhan Textile University, Wuhan 430073, China; 1915043029@mail.wtu.edu.cn

**Keywords:** anti-cancer, reactive oxygen species, chelators, metal complexes, antioxidant enzymes, SOD1, TrxR, mitochondria

## Abstract

Reactive oxygen species (ROS) are rapidly eliminated and reproduced in organisms, and they always play important roles in various biological functions and abnormal pathological processes. Evaluated ROS have frequently been observed in various cancers to activate multiple pro-tumorigenic signaling pathways and induce the survival and proliferation of cancer cells. Hydrogen peroxide (H_2_O_2_) and superoxide anion (O_2_^•−^) are the most important redox signaling agents in cancer cells, the homeostasis of which is maintained by dozens of growth factors, cytokines, and antioxidant enzymes. Therefore, antioxidant enzymes tend to have higher activity levels to maintain the homeostasis of ROS in cancer cells. Effective intervention in the ROS homeostasis of cancer cells by chelating agents or metal complexes has already developed into an important anti-cancer strategy. We can inhibit the activity of antioxidant enzymes using chelators or metal complexes; on the other hand, we can also use metal complexes to directly regulate the level of ROS in cancer cells via mitochondria. In this review, metal complexes or chelators with ROS regulation capacity and with anti-cancer applications are collectively and comprehensively analyzed, which is beneficial for the development of the next generation of inorganic anti-cancer drugs based on ROS regulation. We expect that this review will provide a new perspective to develop novel inorganic reagents for killing cancer cells and, further, as candidates or clinical drugs.

## 1. Introduction

**Figure 1:**
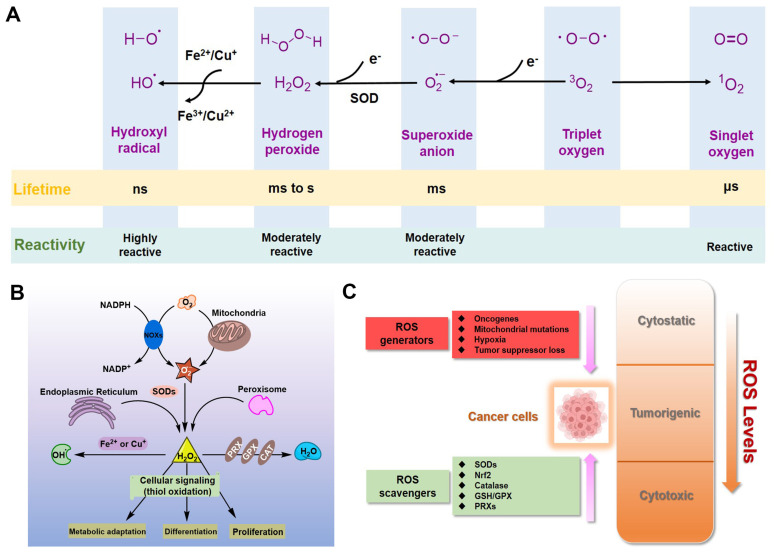
ROS in cancer cells. (**A**) Generation and chemical structures of ROS. (**B**) Brief metabolic process and signal regulation of intracellular ROS. (**C**) Balancing ROS generation and scavenging in cancer cells to remain in the tumorigenic range.

For eukaryotic cells, reactive oxygen species (ROS) encompass a group of molecules derived from oxygen, such as hydrogen peroxide (H_2_O_2_), superoxide anion (O_2_^•−^), organic hydroperoxides (ROOH), singlet molecular oxygen (^1^O_2_), hydroxyl radical (•OH), alkoxyl radical (•OR), and peroxyl radical (•OOR) [1,2,3]. ROS are mainly formed by reduction–oxidation reactions or by electronic excitation (Figure 1A) [1,2,3] and have evolved as regulators of multiple signaling pathways [4,5,6,7,8]. Two species, H_2_O_2_ and O_2_^•−^, are the most important redox signaling agents in the cells [4,5,6,7,8]. H_2_O_2_ is the major ROS in organisms, with its concentration always maintained within 1~100 nM under normal conditions [8]. For O_2_^•−^, the concentration is also maintained at about 0.01 nM, much lower than that of H_2_O_2_ [8].

Dozens of growth factors, cytokines, and antioxidant enzymes control the homeostasis of intracellular H_2_O_2_ and O_2_^•−^ (Figure 1B) [9]. O_2_^•−^ is prominently generated by the mitochondrial electron transport chain (Mito-ETC), NADPH oxidase (NOX) complex, and endoplasmic reticulum (ER) system, and is rapidly converted to H_2_O_2_ by superoxide dismutases (SODs) [10,11,12]. Subsequently, H_2_O_2_ is mainly detoxified to H_2_O by catalase (CAT), glutathione peroxidase (GPX), and peroxiredoxin (Prx) [12]. It is worth mentioning that •OH formed by metal-catalyzed Fenton reaction is the most reactive ROS; it can oxidize biological macromolecules indiscriminately, such as DNA, proteins, and lipids [12]. Therefore, maintaining the homeostasis of intracellular ROS is essential for cell growth, proliferation, and survival [6,9,10].

Due to the well-established role of ROS in cell signaling, cancer cells always have higher levels of endogenous ROS to enhance rapid cell growth and proliferation through the mitogen-activated protein kinase (MAPK)/extracellular-regulated kinase 1/2 (ERK1/2), phosphoinositide-3-kinase (PI3K)/Akt, nuclear factor-κB (NF-κB), and hypoxia-sensitive α (HIF1α) pathways [13,14,15,16,17,18]. Indeed, higher levels of ROS have already been observed in various cancer cells [11,19]. If the intracellular ROS levels increase dramatically to toxic concentrations, oxidative stress will cause irreversible damage and may eventually lead to the death of cancer cells [11,20]. To maintain the elevated mitogenic signaling without incurring substantial oxidative damage by a proper balance of ROS, the antioxidant enzymes in cancer cells, such as Cu/Zn superoxide dismutase (SOD1), GPX, and Prx, should harbor higher levels of activity (Figure 1C) [21,22,23,24].

Elevated levels of ROS are always involved in the initiation and progression of cancer. Hence, intervening in the homeostasis of ROS in cancer cells is an effective anti-cancer strategy [25,26]. So far, a variety of chelators or metal complexes based on the regulation of ROS have been reported as anti-cancer agents [27,28,29,30,31,32,33,34]. For those antioxidant enzymes where the active center is a metal ion, chelators can be used to competitively bind to the metal ion and thus inhibit the enzymatic activity to achieve the regulation of intracellular ROS, including SOD1 inhibitors tetrathiomolybdate (**ATN-224**) and **LD100** [32,33]. On the other hand, several metal complexes can regulate the ROS levels in cancer cells through other mechanisms to achieve anti-cancer purposes, such as TxrR inhibition and mitochondrial dysfunction [34,35,36]. Regulating the relative level of ROS in cancer cells through the mechanism of metal coordination has become an important branch with broad prospects in the field of cancer therapy.

In this review, chelators or metal complexes with ROS regulation capacity and with anti-cancer applications are collectively and comprehensively analyzed [32,33,34,35,36,37,38]. The strategy based on coordination chemistry to regulate the level of intracellular ROS has developed into an important method to kill cancer cells. We expect that further study of ROS regulation by metal coordination will provide a new perspective to develop novel reagents for killing cancer cells and, further, as candidates or clinical drugs for cancer therapy.

## 2. Inorganic SOD1 Inhibitors with Anti-Cancer Prospects

In mammals, the main biological function of SODs is to catalyze the dismutation of O_2_^•−^ into H_2_O_2_ and O_2_ [39,40]. Cu/Zn superoxide dismutase (SOD1), the major SOD, mainly exists in the formation of homodimers in cells and is widely distributed in the nucleus, the cytoplasm, and the intermembrane space (IMS) of mitochondria [41]. Next, Mn superoxide dismutase (SOD2) exclusively exists in the mitochondrial matrix [42]. An extracellular form of SOD (EC-SOD), also a Cu/Zn-containing SOD, is tetrameric and exists in most mammals [42]. Besides this, SOD1 also regulates multiple redox signals to control growth and metabolic pathways, such as glucose metabolism and transcription [5,43,44,45]. Therefore, SODs, especially SOD1, are the first firewall to resist oxidative stress.

Recently, emerging evidence from researchers has indicated that SOD1 is usually overexpressed in cancer cells; its activity is essential to maintain higher ROS levels under the critical threshold during aberrant energy metabolism of cancer progression [41]. For example, SOD1 accumulations were observed not only in the cytoplasm but also in the nucleus of human primary breast and mammary cancers [46]. Besides this, prostate cancer cells (DU145) also have higher levels of activity and expression of SOD1, compared with normal prostate cells (RWPE-1) [5]. In vitro studies also showed that the fast growth of non-small cell lung cancer (NSCLC) and leukemia depends on the high activity of SOD1, which controls the oncogenic KRAS and EGFR pathways [47,48], as well as other cancer cells and xenograft tumors [49]. In general, SOD1 is recognized as a promising anti-cancer target, and several small-molecule targeting drugs for SOD1 have already entered the preclinical and clinical development stages [50].

**Figure 2:**
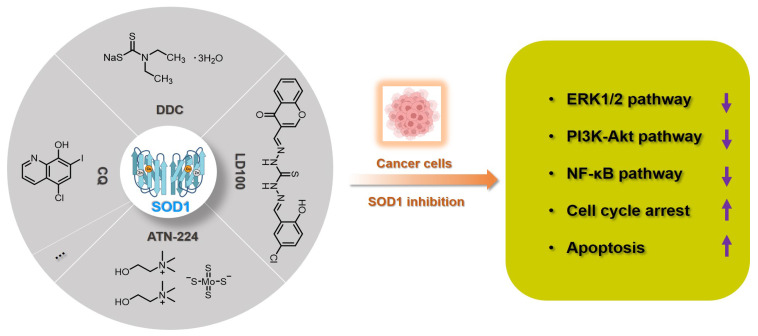
Summary of SOD1 inhibitors based on copper chelation. The specific activity inhibition of SOD1 selectively kills cancer cells by regulating the intracellular ROS signaling network.

Since the activity of SOD1 mainly comes from the copper ion in the active center, a vast majority of SOD1 inhibitors are competitive chelators of copper ions. In 1975, Heikkila et al. found that diethyldithiocarbamate (**DDC**) can competitively bind to copper ions (Figure 2), thereby inhibiting SOD1 activity at a millimolar level [51]. After being inhibited by **DDC**, SOD1 cannot restore enzyme activity through dialysis, but adding CuSO_4_ during dialysis restores SOD1 activity [51]. In 1979, Misra systematically explored the mechanism by which **DDC** inhibits SOD1 activity [52]. In Phase I, one **DDC** molecule first coordinates with the copper(II) center in SOD1, with retention of activity. In Phase II, a second **DDC** displaces the copper(II) center, with a loss of activity. The shortcomings of **DDC** as a SOD1 inhibitor are mainly reflected in its high working concentration and poor specificity, such as its interference with the activity of cytochrome *c* oxidase [53,54]. Nevertheless, **DDC** still has a wide range of anti-cancer applications, and **DDC** effectively inhibits SOD1 activity to kill cancer cells [55,56,57].

In 2005, Ding et al. found that clioquinol (5-chloro-7-iodo-8-hydroxyquinoline, **CQ**), a metal chelator of copper/zinc/iron, is another SOD1 inhibitor (Figure 2), because the copper and zinc ions in the active sites of SOD1 are coordinated by **CQ** [58]. Structural characterization of the zinc(II) and copper(II) complexes with **CQ** indicated that the stoichiometry of ligand to metal is 2:1 [59]. Therefore, **CQ** can effectively inhibit SOD1 at micromolar concentrations (IC_50_: 6.7~43.1 μM) and induce the death of a variety of cancer cells through the caspase-3-mediated apoptosis pathway [58]. It cannot be ignored that **CQ** has the risk of destroying copper homeostasis during its inhibition of SOD1 activity in cells [60].

Tetrathiomolybdate is an orally available copper chelator that has been shown to have efficacy as an anti-angiogenic and anti-tumor agent in multiple cancers [61,62]. **ATN-224** is the second-generation choline salt of tetrathiomolybdate with improved performance (Figure 2) and is being evaluated in several phase II trials in cancer patients [63]. Doñate et al. found that **ATN-224** can selectively bind copper with high affinity, and SOD1 is the main target for the anti-angiogenic activity of this chelator [32,64]. **ATN-224** also inhibits intracellular SOD1 activity at micromolar concentrations (IC_50_: 1.4~185 μM), but has specificity for copper binding, all of which makes it one of the most popular SOD1 inhibitors [62,64]. Every sulfur atom in tetrathiomolybdate can coordinate with copper and may then form metal clusters with copper enzymes, thereby inhibiting the activity of copper proteins, such as SOD1, cytochrome *c* oxidase, and ceruloplasmin [65,66]. Therefore, **ATN-224** may also interfere with intracellular copper homeostasis or inhibit other copper enzymes. In cancer treatment, **ATN-224**-mediated SOD1 inhibition led to the downregulation of PDGF and increase of O_2_^•−^, prevented the formation of high levels of H_2_O_2_, and protected protein tyrosine phosphatases from oxidation by H_2_O_2_ [62]. Therefore, SOD1 inhibition by **ATN-224** results in the down-regulation of multiple signaling pathways for cancer cell function, such as ERK1/2 and anti-apoptotic factor Mcl1 [50,62].

Considering that the known SOD1 inhibitors have various defects, such as low efficiency, weak specificity, and interference with the homeostasis of metal ions, we designed a next-generation SOD1 inhibitor (**LD100**) based on copper coordination chemistry and the catalytic cycle in the active site (Figure 2) [33]. **LD100** was designed through the combination of thiosemicarbazone and phenol derivatives, because thiosemicarbazone contains a copper chelating moiety, -C(SH)-NH-, and the phenolic hydroxyl can further facilitate the copper coordination. Besides this, **LD100** also contains a fluorescent group chromone, which not only can be used to track the entry of **LD100** into cells, but also enables **LD100** to better occupy the substrate channel of SOD1. Therefore, **LD100** has a strong binding ability to copper ions in solution and can effectively inhibit the activity of SOD1 in vitro and in vivo (IC_50_ of **LD100** to SOD1 in HeLa cells: 0.18 μM) [33]. Through specific inhibition of SOD1 activity, **LD100** can efficiently up-regulate the intracellular concentration of O_2_^•−^, down-regulate the concentration of H_2_O_2_, down-regulate the phosphorylation of ERK1/2, and finally induce the apoptosis of cancer cells [33]. In summary, **LD100** may be the most effective SOD1 inhibitor so far and has application prospects for cancer treatment. Using this inhibitor, we also systematically explored the mechanism of how SOD1 activity inhibition selectively kills cancer cells [5]. The rapid growth and proliferation of cancer cells always depend on higher SOD1 activity, so cancer cells are more sensitive to SOD1 inhibition. During SOD1 inhibition in cancer cells, LD100 could repress the ERK, PI3K-Akt, and NF-κB pathways; arrest the cell cycle; and induce mitochondria-dependent apoptosis [5].

SOD1 is indeed a recognized target for cancer treatment. At present, a variety of chelators have been used for SOD1 inhibition. **LD100** may be the most effective inhibitor designed through coordination chemistry. However, the use of inorganic strategies to develop anti-cancer drugs based on SOD1 inhibition still requires further efforts. First, we need to solve the problem of compatibility between targeted and clinical deliveries. On the other hand, we also should reduce the side effects of chelating agents while ensuring the efficiency of SOD1 inhibition. The summary of SOD1 metal-chelating inhibition can provide a reference for the design of SOD1 inhibitors with anti-cancer effects in the future.

## 3. Anti-Cancer Metal Complexes Inhibiting Antioxidant Enzymes

**Figure 3:**
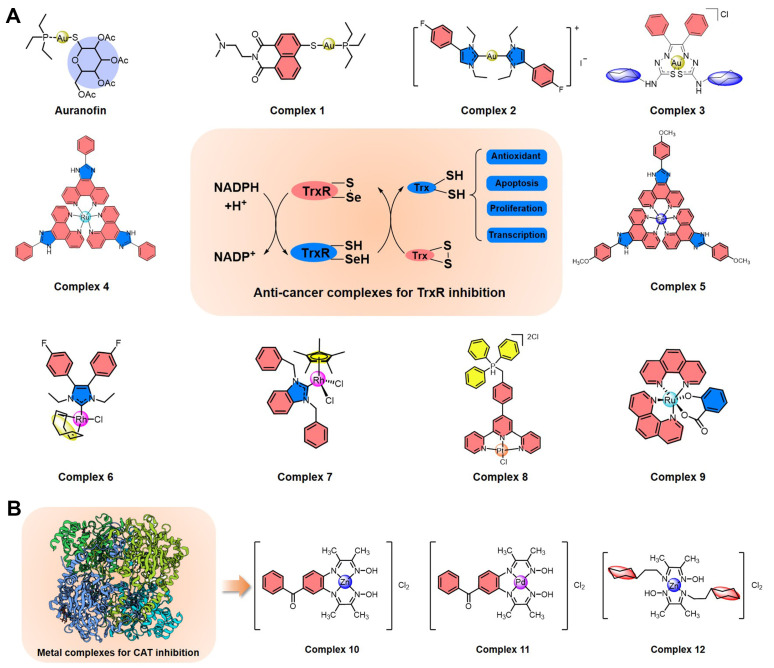
Summary of metal complexes inhibiting TrxR and CAT. (**A**) Structures of anti-cancer metal complexes for TrxR inhibition to regulate intracellular ROS levels. (**B**) Structures of metal complexes for CAT inhibition.

Compared to normal cells, cancer cells always harbor higher levels of ROS and antioxidant enzymes to induce uncontrolled proliferation and a high metabolic rate [67]. In addition to the aforementioned chelators used to inhibit SOD1 activity, a variety of metal complexes targeting other members of antioxidant enzyme systems have also been developed. In higher organisms, thioredoxin (Trx) and thioredoxin reductase (TrxR) are the core members of the Trx system and have also been recognized as another key modulator of cancer development [68]. The main function of TrxR is to reduce the oxidized disulfide of Trxs to the reduced dithiol form by taking electrons for NADPH, and high TrxR activity is also crucial for cancer cells (Figure 3A) [68]. According to the Hard and Soft Acids and Bases (HSAB) theory, sulfur/selenium of TrxR (soft base) should have a high affinity for gold and other noble metals (soft acid). Hence, the use of metal complexes to inhibit the activity of TrxR has also been developed as an anti-cancer strategy, by reducing the level of reduced Trx in cancer cells [68,69,70,71].

As early as 1998, Schirmer et al. first found that auranofin (Figure 3A), an organic gold compound with anti-cancer activity, can inhibit TrxR in the nanomolar range through the formation of a covalent interaction between the gold atom and disulfide bond [72,73]. Inspired by TrxR inhibition by auranofin, Ott et al. developed another gold(I) phosphine complex (**1**) (Figure 3A) [74]. The ligand of **1** contains a pharmacophore of the naphthalimide class with anti-cancer activity, including a heterocyclic naphthalimide core for DNA intercalation and a side chain containing a protonable nitrogen for DNA phosphate backbone contraction. In cancer cells, **1** can effectively inhibit TrxR activity, suppress rapid proliferation, and promote mitochondrial-dependent apoptosis. This complex is also enriched in the nucleus of tumor cells, possibly exerting the anti-cancer activity of naphthalimide. In 2019, Ott et al. used another gold(I) biscarbene complex (**2**) (Figure 3A) with an N-heterocyclic carbene ligand to improve the inhibition efficiency of TrxR and to enhance the antiproliferative effects of cancer cells [75]. Besides this, Pizarro et al. also synthesized a gold(III) benzil bis(thiosemicarbazonate) complex (**3**) (Figure 3A) that can be enriched in the cytoplasm and mitochondria of MCF-7 cells and effectively inhibit TrxR activity, leading to a dramatic alteration of the cellular redox state and to the induction of cell death [76]. Of course, there are also various other gold complexes with anti-cancer prospects that can effectively inhibit the activity of TrxR.

In addition to gold complexes, a variety of other metal (platinum, ruthenium, rhodium, iridium, iron, palladium, silver) complexes also inhibit TrxR and may have anti-cancer potential. In 2014, Chen et al. developed a ruthenium polypyridyl complex (**4**) as an inducer of ROS-mediated apoptosis in cancer cells by targeting TrxR (Figure 3A) [37]. This complex can effectively inhibit TrxR at the micromolar level within a few minutes and can suppress cancer cell growth through cell cycle arrest and induction of apoptosis. Furthermore, an iron(II) complex (**5**) with a phenanthroline derivative as a ligand was also reported by Chen et al. in 2016 (Figure 3A) [77], with inhibitory efficiency for TrxR improved by at least an order of magnitude compared with that of **4**. Complex **5** also kills cancer cells by ROS-mediated apoptosis by targeting TrxR, and it further improves compatibility in animal models [37]. Next, rhodium complexes are also a large class of conventional TrxR inhibitors, such as rhodium(I) complex **6** and rhodium(III) complex **7** [78,79], all of which can kill cancer cells by TrxR-inhibition-mediated ROS regulation (Figure 3A). Recently, Chen et al. also found that a novel triphenylphosphonium-modified terpyridine platinum(II) complex (**8**) is an inhibitor of mitochondrial TxrR with enhancement of caspase-3-independent apoptosis by increasing cellular ROS [80]. Besides this, a ruthenium(II) salicylate complex (**9**) with anti-cancer potential was developed by Lan-mei Chen et al., which can selectively kill cancer cells, induce apoptosis, trigger cell cycle arrest and DNA damage, and promote the accumulation of ROS by specific TrxR inhibition [81]. Overall, metal complexes based on TrxR inhibition regulate the level of reduced Trx, destroy ROS homeostasis, and then kill cancer cells by apoptosis.

In addition, a few metal complexes have been developed to target other antioxidant enzymes such as Prx and CAT. Wang et al. demonstrated that a ruthenium complex [(η^6^-arene)Ru(en)Cl]^+^ can inhibit the enzymatic activity of human peroxiredoxin I through coordination with the catalytic site Cys173 [82]. However, due to the numerous subtypes of intracellular Prxs, it is difficult to design highly efficient and targeted metal complexes. It is worth mentioning that Shahraki et al. recently developed several potential anti-cancer metal complexes with the potential to inhibit CAT (Figure 3B) [83,84,85]. For example, both zinc(II) complex **10** and palladium(II) complex **11** can bind to the CAT enzyme cavity and alter the structure and conformation of CAT [83]. Besides this, zinc(II) complex **12** with a novel bidentate Schiff base ligand can also inhibit CAT by the conformational changes from van der Waals forces and hydrogen bonds; it also has anti-cancer activity in human colon cancer cells [84].

Contributions from the Ott group, Chen group, Shahraki group, and others have greatly enriched the entry of metal complexes targeting the antioxidant enzyme system as promising anti-cancer agents [74,75,76,77,78,79,80,81,82,83,84,85]. Despite the importance of metal complexes as antioxidant enzyme inhibitors, no clinical anti-cancer drug targets TrxR, CAT, or Prx currently, which may be due to several major challenges in the field. For example, the metal complexes designed by targeting cysteine have the risk of non-specific effects. We may also need to improve the efficiency of metal complexes in inhibiting antioxidant enzymes and reduce the interference of ROS homeostasis in normal cells during drug treatment. The summary of anti-cancer metal complexes targeting the antioxidant enzyme systems in this chapter is expected to give inspiration for anti-cancer drug design, which might potentiate the clinical application of metal complexes in cancer treatment.

## 4. Anti-Cancer Metal Complexes Activating ROS-Mediated Signaling from Mitochondria

Functional mitochondria are essential for higher energy supply to cancer cells through oxidative phosphorylation [86], and they control various vital cellular parameters, including ATP production, oxidation–reduction status, ROS, cytosolic calcium and biosynthetic precursor levels, oncogenic signaling, innate immunity, and apoptosis through the activation of mitochondrial permeability transition pores [86,87,88]. In cancer cells, the metabolism is reprogrammed for energy supply from oxidative phosphorylation to aerobic glycolysis [89,90], and mitochondrial biogenesis and quality control are always upregulated [91,92].

ROS overproduction in the mitochondria of cancer cells promotes cancer progression by increasing genomic instability, regulating gene expression, and controlling multiple signaling pathways [91,92]. On the other hand, oxidative damage to mitochondria and mitochondrial DNA impairs the oxidative phosphorylation process and results in further ROS production, which forms a vicious cycle involving ROS, mitochondria, genomic instability, and cancer deterioration [93,94,95]. Hence, the major contributor to cancer development from mitochondria is ROS, especially from dysfunctioning or malfunctioning mitochondria [94]. So far, regulating the function of mitochondria in cancer cells to control the level of intracellular ROS has already been developed as a powerful anti-cancer method [94,95]. Compared with normal cells, the mitochondria of cancer cells always have a higher membrane potential [96], and the delivery of inorganic drugs into mitochondria based on the higher mitochondrial membrane potential allows selective targeting of cancer cell mitochondria to kill cancer cells [97,98]. Recently, anti-cancer metal complexes targeting mitochondria have drawn strong interest because of their strong anti-cancer activities, limited side effects, and versatile photophysical properties.

**Figure 4:**
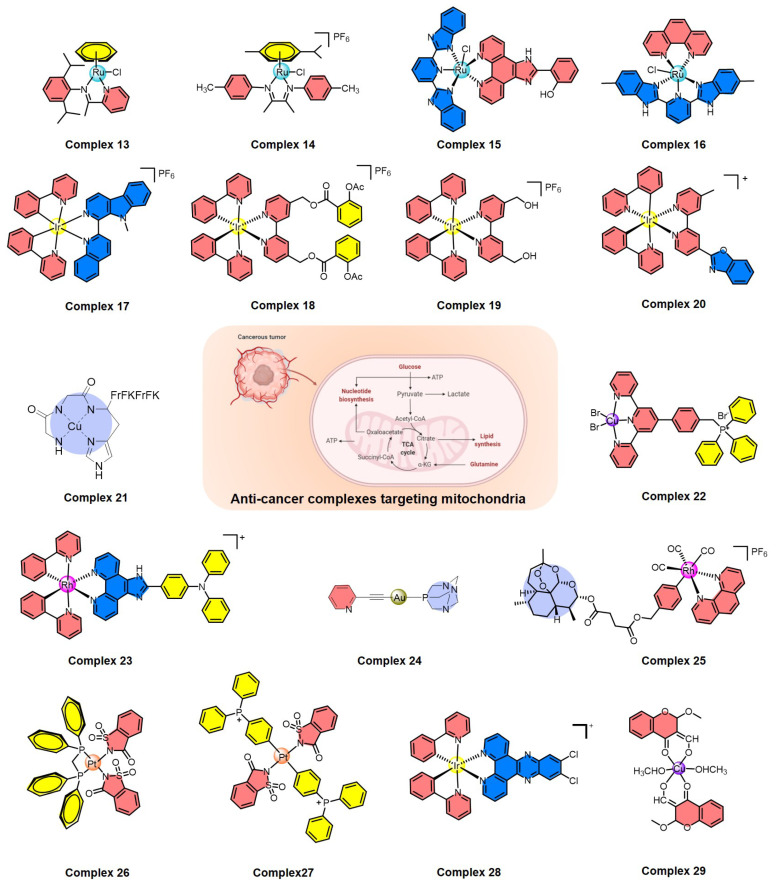
Summary of metal complexes activating ROS-mediated signaling by mitochondrial dysfunction.

In recent years, a variety of ruthenium complexes that act on mitochondria have been developed, and these complexes can often up-regulate intracellular ROS levels and activate ROS-mediated signaling to kill cancer cells (Figure 4). In 2017, Liu et al. reported a half-sandwich ruthenium(II) complex (**13**) with an N^N-chelated imino-pyridyl ligand as a selective anti-cancer agent; it evaluates intracellular ROS, disrupts mitochondrial membrane potential, and then kills A549 cancer cells [99]. They also developed another mitochondria-targeted half-sandwich ruthenium(II) diimine complex (**14**) as an anti-cancer agent via ROS-mediated signaling; **14** can effectively locate in the mitochondria and inhibit the migration of cancer cells [100]. These half-sandwich ruthenium complexes and derivatives have great value for development as novel theranostic candidates due to their mitochondrial imaging and anti-cancer prospects. Of course, the metal complexes acting on mitochondria are not limited to the half-sandwich structure, such as ruthenium polypyridyl complexes. For example, Chen et al. synthesized a mixed-ligand ruthenium polypyridyl complex (**15**) with an *ortho*-phenolic group on the ligand; **15** can enter cancer cells through endocytosis and then translocate from lysosomes to the mitochondria, where **15** activates mitochondrial dysfunction and up-regulates the intracellular ROS level to selectively kill cancer cells [101]. Subsequently, Chen et al. also found that aquation of ruthenium complex **16** can effectively enhance its hydrophilicity and cellular uptake, thus significantly increasing its anti-cancer efficacy by mitochondrial dysfunction [102], which provides valuable information for the rational design of next-generation ruthenium polypyridyl complexes.

In addition, cyclometalated iridium complexes are another type of anti-cancer agent acting on functional mitochondria (Figure 4). Mao et al. reported several representative cyclometalated iridium complexes with anti-cancer activity through loss of mitochondrial membrane potential and elevation of intracellular ROS, such as iridium complexes **17** and **18** [103,104]. For **17**, the ubiquitin-proteasome system (UPS) was also induced and resulted in the collapse of mitochondria and subsequent cytoplasmic vacuolation because of the rapid loss of mitochondrial functions [103]. For **18**, caspase-dependent apoptosis, caspase-independent paraptosis, and metastasis were controlled, and this complex also showed tumor growth inhibition in vivo [104]. Similarly, mitochondria-targeting cyclometalated iridium complexes were also used as potent anti-glioma stem cell agents, such as complex **19** [105]. On the other hand, two iridium complexes were synthesized as necroptosis inducers in cisplatin-resistant cancer cells, such as complex **20**, which can selectively accumulate in mitochondria, disrupt the mitochondrial membrane potential, and circumvent drug resistance, leading to the activation of receptor-interacting serine-threonine kinase 3 (RIPK3) and mixed lineage kinase domain-like pseudokinase (MLKL) [106]. Therefore, the development of iridium-based complexes offers the opportunity to bypass drug resistance and improve the efficiency of killing cancer cells.

Recently, many other metal complexes targeting mitochondria have also been developed to kill cancer cells by regulating the level of intracellular ROS (Figure 4), such as copper complexes **21** and **22** [107,108], rhodium complex **23** [109], gold complex **24** [110], and rhenium complex **25** [111]. Interestingly, **24** is also a necroptosis inducer and a potential option in cases of apoptosis resistance; it disrupts the normal function of mitochondria, leading to ROS elevation in colorectal adenocarcinoma cells [110]. Another thing worth mentioning is that rhenium complex **25** can induce both apoptosis and ferroptosis in cancer cells through mitochondrial dysfunction, caspase cascade, glutathione depletion, glutathione peroxidase 4 inactivation, and lipid peroxidation, which is a promising strategy to induce both apoptosis and ferroptosis at the same time [111].

A variety of metal complexes have been developed that not only act on mitochondria but also can bind to DNA (Figure 4), which can cause excessive DNA damage to kill cancer cells, including platinum complexes **26** and **27** [112,113], iridium complex **28** [114], and copper complex **29** [115]. Taking **27** as an important example, **27** can interact with DNA through groove binding and has the potential to break DNA [113]. Mechanistic studies indicated that this complex causes excessive generation of ROS and displays dual action by targeting both mitochondria and genomic DNA [113]. Therefore, complexes acting on mitochondria and genomic DNA at the same time have broad prospects in the field of designing new non-polar drugs in the future.

## 5. Conclusions

In this review, we systematically summarized the latest advances in developing inorganic chelators and metal complexes as anti-cancer agents through regulating intracellular ROS levels, with a particular focus on those targeting mitochondria and antioxidant enzyme systems, including SOD1, TrxR, and CAT. Metal coordination and metal complexes display a preeminent combination of biological activity, cell permeability, and stability in cancer cells. Using inorganic strategies to destroy the ROS homeostasis in cancer cells is powerful and gives significant prospects for the future application of chelators and metal complexes as anti-cancer drugs.

For the antioxidant enzyme systems, a large number of inorganic inhibitors have been developed, such as SOD1 inhibitors (**DDC**, **CQ**, **ATN-224**, and **LD100**), TrxR inhibitors (auranofin and **1**–**9**), and CAT inhibitors (**10**–**12**). Generally, these chelators or metal complexes can induce apoptosis, arrest the cell cycle, regulate ROS signaling pathways, and kill cancer cells through antioxidant enzyme inhibition and consequent ROS elevation. Although **ATN-224** has entered clinical testing, there is still a lack of inorganic anti-cancer drugs targeting antioxidant enzyme systems. The problems that need to be solved mainly involve the efficiency and specificity of antioxidant enzyme inhibition, the targeting of cancer tissues, and toxic side effects.

Multiple strategies of cancer-selective mitochondrial damage can lead to apoptosis, necroptosis, and ferroptosis by the utilization of metal complexes, such as ruthenium polypyridyl complexes, cyclometalated iridium complexes, and so on (**13–29**). For the high performance of anti-cancer metal complexes acting on mitochondria, several challenges still need to be overcome, including anti-cancer activity, mitochondrial targeting, and toxic side effects. Metal complexes with dual effects on functional mitochondria and genomic DNA may have better application prospects in improving anti-cancer performance. In order to further reduce the side effects, we can improve the efficiency of metal complexes targeting cancer cell mitochondria, such as by conjugation of mitochondria-targeting peptides and DNAs.

The summary of anti-cancer chelators and metal complexes targeting antioxidant enzyme systems or mitochondria in this review is expected to give inspiration for the design of next-generation inorganic anti-cancer drugs. To design novel SOD1 inhibitors, we can further optimize the type of coordination group and adjust the molecular conformation of chelators to match the active cavity of SOD1. For the rational design of anti-cancer metal complexes, we can not only adjust the combinatorial mode of ligands and metal ions, but also apply different metal ions to different systems. Besides this, the development of multifunctional metal complexes may be another direction for the design of anti-cancer drugs, such as the combination of antioxidant enzyme inhibition, mitochondrial destruction, and genomic DNA interaction. In any case, the application of cancer-targeting groups is an optional strategy to reduce off-target effects. In the future, research on the development of inorganic anti-cancer drugs is highly expected to result in clinical trials for efficient and side-effect-free cancer therapeutics.

## Data Availability

Data sharing not applicable.
